# *Claea tongziensis* sp. nov., a new species of *Claea* (Teleostei, Cypriniformes, Nemacheilidae) from the upper Yangtze River drainage, China

**DOI:** 10.3897/zookeys.1284.187814

**Published:** 2026-07-10

**Authors:** Meng-Fei Zhao, Hai-Min Lv, Jun-Hao Huang, Fei Liu

**Affiliations:** 1 Institute of Hydrobiology, Chinese Academy of Sciences, No. 7 Donghu South Road, Wuchang District, Wuhan 430072, Hubei Province, China Institute of Hydrobiology, Chinese Academy of Sciences, No. 7 Donghu South Road, Wuchang District Wuhan China; 2 University of Chinese Academy of Sciences, No. 1 Yanqi Lake East Road, Huairou District, Beijing 100049, China University of Chinese Academy of Sciences Beijing China

**Keywords:** Chishui River, morphology, phylogeny, taxonomy

## Abstract

A new species of the genus *Claea*, namely *Claea tongziensis***sp. nov**., is described from the Tongzi River, a tributary of the Chishui River, in Tongzi County, Guizhou Province, China. *Claea tongziensis***sp. nov**. is morphologically distinguished from its congeners by the combination of the following characters: seven branched dorsal-fin rays; three unbranched anal-fin rays; vertebrae 4+40–42; prominent processus dentiformis; prepelvic length 46.9–51.7% SL; pelvic–anal distance 23.8–28.6% SL; anus–anal distance 4.7–7.0% SL; eye diameter 13.5–18.5% HL; interorbital width 22.2–29.6% HL; outer rostral barbel length 18.9–29.3% HL. In molecular analyses, *C. tongziensis***sp. nov**. constitutes a monophyly in a phylogenetic tree based on mitochondrial *cytb* and *cox1* gene sequences and is significantly distant from its congeners in genetic distance (2.82–6.41%) based on the *cytb* gene. Morphological and molecular evidence support the validity of the new species.

## Introduction

**Figure 1.**
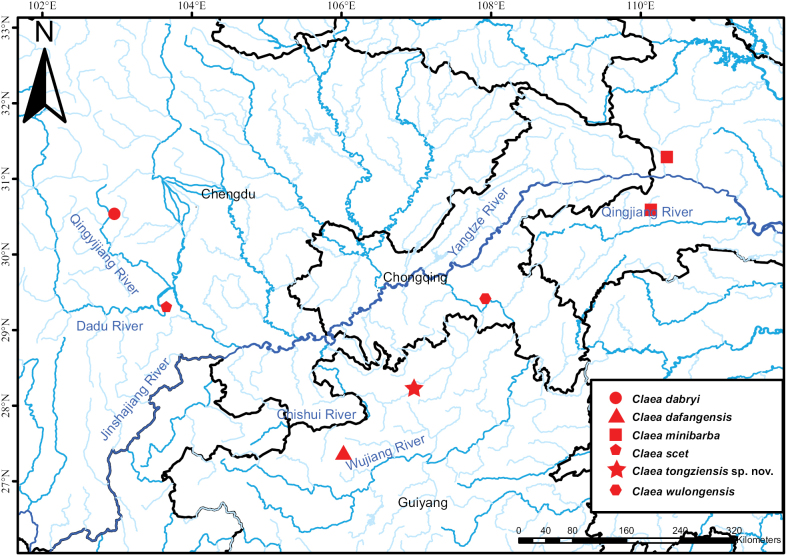
The type locality of *Claea tongziensis* sp. nov., and five previously described species of *Claea*.

The genus *Claea* Kottelat, 2011 are small benthic loaches in the family Nemacheilidae (Cypriniformes) and are adapted to mountain streams with flowing water and gravel substrates. *Claea* can be morphologically distinguished from other genera of Nemacheilidae based on the following diagnostic characters: body smooth and scaleless; lateral line fully developed; anterior and posterior nostrils closely set; nasal barbels absent; processus dentiformis at the middle of the upper jaw; swim bladder dumbbell-shaped with the anterior chamber bony and the posterior chamber degenerate; no sexual dimorphism in breeding tubercles on the sides of the head and on the dorsal surfaces of broadened pectoral fins; adipose crest absent along dorsal and ventral midlines of the caudal peduncle; and supra-pelvic flap absent (Zhang et al. 2024; Lei et al. 2025). However, none of these characters are unique to *Claea*. Species of *Claea* have suffered a complex taxonomic history. The genus was originally erected as *Oreias* by Sauvage (1874) to accommodate the newly described species *Oreias dabryi* Sauvage, 1874 from Qingyijiang River, Sichuan Province. Subsequent taxonomic treatments reclassified *O. dabryi* as belonging to the genera *Barbatula* Linck, 1790 and *Nemachilus* Günther, 1868 owing to inadequate original diagnoses and ambiguous type localities (Nichols 1944; Cao and Wu 1962). By 1988, two new species, *O. crassipedunculatus* Bănărescu & Nalbant, 1976 and *O. furcatus* Bănărescu & Nalbant, 1976, and one new subspecies, *O. dabryi nanpanjiangensis* Zhu & Cao, 1988 had been successively described (Bănărescu and Nalbant 1976; Zhu and Cao 1988). Zhu (1989) later proposed the synonymy of *Oreias* under *Schistura* McClelland, 1838, subsuming *O. furcatus* and *O. crassipedunculatus* into *S. dabryi*. However, the validity of *Oreias* was restored by Chu and Chen (1990), and *O. dabryi nanpanjiangensis* was transferred to *Triplophysa* Rendahl, 1933 based on the absence of a prominent processus dentiformis at the medium of the upper jaw and the presence of sexual dimorphism, which are diagnostic features of *Triplophysa* (Zhu 1989). Later, *S. dabryi microphthalmus* Liao & Wang, 1997 and *S. niulanjiangensis* Chen, Lu & Mao, 2006 (Liao et al. 1997; Chen et al. 2006) were described. The genus *Claea* was eventually established by Kottelat (2011) as a replacement for the preoccupied name *Oreias*. In a subsequent revision, Kottelat (2012) reassigned *S. dabryi microphthalmus* to *Triplophysa* and *S. niulanjiangensis* to *Claea*. Chen et al. (2021) described a new cave-dwelling species, *T. wulongensis* Chen, Sheraliev, Shu & Peng, 2021, from a subterranean pool, connected to the Wujiang River in Chongqing, China, despite lacks definitive troglobitic adaptations. Zhang et al. (2024) reclassifed *T. wulongensis* as *Claea*, transferred *C. niulanjiangensis* to *Triplophysa* by examining specimens, and described a new species, *C. minibarba* Zhang, Luo, Huang & Zhang, 2024, from the Shennongxi and Qingjiang River in Hubei Province, China. Lei et al. (2025) described the first obligate cave-dwelling species of *Claea*, *C. scet* Lei, He, Huang, Zhou & He, 2025, from the Taojin Cave, a subterranean tributary of the Dadu River in Sichuan Province, China. Wang et al. (2026) described a new cave-dwelling fish, *C. dafangensis* Wang, Luo, Xiao, Xie, Wang, Deng, Xiao & Zhou, 2026, from the Yuchong River, a tributary of the Wujiang River in Guizhou Province, China, which also lacks typical troglobitic adaptations. Currently, five species of *Claea* have been formally described: *C. dabryi*, *C. wulongensis*, *C. minibarba*, *C. scet*, and *C. dafangensis*. And all of them are known to be distributed in the mid-upper reaches of the Yangtze River drainage in China (Fig. 1).

Free of dams on its mainstream, the Chishui River is the only free-flowing tributary in the upper Yangtze River drainage and serves as an important component of the National Nature Reserve for Rare and Endemic Fishes of the upper Yangtze River (Wu et al. 2011; Liu et al. 2020). This river, located in southwestern China, traverses the provinces of Yunnan, Guizhou, and Sichuan. Its upper and middle reaches are characterized by deeply incised valleys, rapid flow, and a dense network of underground rivers and caves, representing a typical karst landscape; the lower reach features a wide and deep channel with gentle flow, indicative of a Danxia landform (Li et al. 2021; Chen et al. 2023). Because of its natural hydrological regime, critical ecological strategic status, and complex geomorphic features, the Chishui River has become an important area for fish diversity research and conservation in the upper Yangtze River drainage. During ichthyological surveys in the Chishui River in November 2023 and May 2024, we collected some specimens referrable to *Claea* from the Tongzi River, a tributary of the middle Chishui River in Guizhou Province. Based on morphological examination and molecular phylogenetic analysis of mitochondrial cytochrome *b* (*cytb*) and cytochrome oxidase subunit 1 (*cox1*) genes, these specimens represented a new species of *Claea*. Here, we formally describe this new species, *Claea tongziensis* sp. nov.

## Materials and methods

### Sample collection

All specimens were collected using a dipnet from the Tongzi River, located in Tongzi County, Guizhou Province, China (Fig. 1). Tissue samples from the right pelvic fin used for molecular analysis were preserved in 95% ethanol. Some specimens used for morphological examination were preserved in 75% ethanol and others in 10% formalin. All collected specimens were stored in the Museum of Aquatic Organisms at the Institute of Hydrobiology (**IHB**), Chinese Academy of Sciences, Wuhan, Hubei Province, China.

### Morphological data acquisition and comparisons

Morphological data were collected from 24 well-preserved specimens. Morphometric and meristic analyses were conducted in accordance with Kottelat (1990) and Chen et al. (2019). The internostril width was measured as the straight-line distance between the upper edge of left and right posterior nostrils. All morphometric measurements were made point-to-point with dial callipers to an accuracy of 0.01 mm. Measurement and counting were done mostly on the left side of specimens. All measurements were presented as percentages relative to the standard length (SL), head length (HL) or caudal peduncle length (CPL). Vertebrae of four specimens were counted from images of microcomputed tomography (micro-CT) reconstructed in CTvox v. 3.3.1 software. Two specimens were dissected to examine the morphological structure of the intestine and swim bladder. Specimens of *C. dabryi*, *C. minibarba*, and *C. scet* from their type localities and used for morphological comparisons were deposited in IHB; specimens of *C. wulongensis* were deposited in the Southwest University School of Life Sciences (**SWU**), Chongqing, China; specimens of *C. dafangensis* were stored in the Guizhou Normal University School of Life Sciences (**GZNU**), Guiyang, Guizhou Province, China.

### DNA extraction, PCR, sequencing, and molecular analyses

Genomic DNA was extracted using the salt-extraction protocol from pelvic fin tissue preserved in 95% ethanol (Peng 2005). The *cytb* and *cox1* genes were amplified by PCR. The primers for *cytb* were L14724 (5’–GACTTGAAAAACCACCGTTG–3’) and H15915 (5’–CTCCGATCTCCGGATTACAAGAC–3’), and the primers for *cox1* were *cox1*-F (5’–CCTACCTGTGGCAATCACRCGC–3’) and *cox1*-R (5’–GTGAATAGGGGGAATCAGTG–3’) (Xiao et al. 2001; Liu et al. 2012). The total reaction system for PCR amplification was 20 μL, comprising 0.5 μl of each forward and reverse primer (10 μM), 1 μl of genomic DNA template, 10 μl of 2×Taq master mix, and 8 μl of dd H_2_O. PCR amplification conditions followed Yan (2017). The products were sequenced at Sangon Biotech (Shanghai) Co., Ltd. All sequences were assembled and corrected using SeqMan program in DNASTAR Lasergene v. 7.1 software package.

The *cytb* and *cox1* gene sequences from four specimens of *Claea tongziensis* sp. nov. were used for phylogenetic analysis, together with two new *cytb* and *cox1* gene sequences of *C. dabryi* from its type locality, along with 35 complete mitochondrial genome sequences and *cytb* and *cox1* gene sequences from the genera *Claea*, *Triplophysa* and *Barbatula* retrieved from GenBank. *Barbatula nuda* (Bleeker, 1865) and *B. toni* (Dybowski, 1869) were selected as the outgroups as phylogenetic analysis revealed that the genus is closely related to *Claea* and *Triplophysa* (Du et al. 2021). The relevant information of all sequences is presented in Table 1. Sequence alignments were performed using MAFFT v. 7.505 (Katoh and Standley 2013) with default settings in PhyloSuite v. 1.2.3 (Zhang et al. 2020; Xiang et al. 2023), then terminal regions were manually trimmed. The Bayesian-inference (BI) and maximum-likelihood (ML) methods were used to construct the phylogenetic trees in PhyloSuite v. 1.2.3. The best-fit nucleotide substitution models for BI and ML phylogenetic trees selected by ModelFinder v. 2.2.0 (Kalyaanamoorthy et al. 2017) under the Akaike information criterion (AIC) were GTR+F+I+G4 and GTR+F+R3, respectively. The BI tree was implemented in MrBayes v. 3.2.7a (Ronquist et al. 2012). Four Monte Carlo Markov Chains (MCMC) were run simultaneously, with two independent runs of 2 million generations, sampling every 1,000 generations. After discarding the initial 25% of samples as burn-in, the remaining ones were used to generate the consensus tree. The ML tree was conducted in IQ-TREE v. 2.2.0 (Nguyen et al. 2015) for 1,000 ultrafast (Minh et al. 2013) bootstraps. Branch credibility of each node in phylogenetic tree was assessed by Bayesian posterior probabilities (BPP) and ML ultrafast bootstrap values (UBP). Final phylogenetic tree visualization and editing were conducted using the online tool Interactive Tree Of Life (iTOL) (https://itol.embl.de/) (Letunic and Bork 2024) and Adobe Illustrator 2025. The mean Kimura 2-parameter (K2P; Kimura 1980) genetic distances based on *cytb* gene sequences between the different species of *Claea* were calculated with 1,000 bootstrap replicates in MEGA7 (Kumar et al. 2016).

**Table 1.** Species used in this phylogenetic analysis with their localities, voucher ID and GenBank accession numbers. Localities are in China, except where otherwise noted.

**Species**	**Localities**	**Voucher ID**	**GenBank accession numbers**	
			***cytb***	***cox1***
*Barbatula nuda*	Irtysh River, Xinjiang	—	KF574248	KF574248
*Barbatula toni*	Amur River, Russia	—	AB242162	AB242162
*Claea dabryi*	Dong River, Sichuan	IHB 20260131	PX896215	PX899326
*Claea dabryi*	Dong River, Sichuan	IHB 20260132	PX896216	PX899327
*Claea dafangensis*	Wujiang River, Guizhou	GZNU20241114008	PX703773	—
*Claea dafangensis*	Wujiang River, Guizhou	GZNU20190804010	PX703774	—
*Claea minibarba*	Qingjiang River, Hubei	IHB 202204287749	OP750011	—
*Claea minibarba*	Qingjiang River, Hubei	IHB 202204287748	OP750012	—
*Claea minibarba*	Qingjiang River, Hubei	IHB 202204287747	OP750013	—
*Claea minibarba*	Qingjiang River, Hubei China	IHB 2017097699	OP750014	—
*Claea minibarba*	Qingjiang River, Hubei	IHB 2017097698	OP750015	—
*Claea scet*	Dadu River, Sichuan	IHB 202305300003	PQ860813	PQ860770
*Claea scet*	Dadu River, Sichuan	IHB 202305300005	PQ860814	PQ860771
*Claea tongziensis* sp. nov.	Chishui River, Guizhou	IHB 202311020001	PX837167	PX837171
*Claea tongziensis* sp. nov.	Chishui River, Guizhou	IHB 202405240003	PX837168	PX837172
*Claea tongziensis* sp. nov.	Chishui River, Guizhou	IHB 202405240008	PX837169	PX837173
*Claea tongziensis* sp. nov.	Chishui River, Guizhou	IHB 202405240011	PX837170	PX837174
*Claea wulongensis*	Wujiang River, Chongqing	—	MW582823	—
*Claea wulongensis*	Wujiang River, Chongqing	GZNU 20230404008	OQ754129	—
*Triplophysa angeli*	—	—	NC065113	NC065113
*Triplophysa anlongensisi*	Nanpanjiang River, Guizhou	T24	PP204904	PP204904
*Triplophysa baotianensis*	Nanpanjiang River, Guizhou	GZNU 20180421005	NC056365	NC056365
*Triplophysa bleekeri*	Daning River, Chongqing	—	JX135578	JX135578
*Triplophysa cehengensis*	Beipanjiang River, Guizhou	T21	PP204902	PP204902
*Triplophysa erythraea*	Yuanjiang River, Hunan	—	PQ040451	PQ040451
*Triplophysa hsutschouensis*	—	—	OR730670	OR730670
*Triplophysa huapingensis*	Hongshui River, Guangxi	—	NC086804	NC086804
*Triplophysa longliensis*	Hongshui River, Guizhou	—	NC086805	NC086805
*Triplophysa markehenensis*	—	—	KT213594	KT213594
*Triplophysa nanpanjiangensis*	Nanpanjiang River, Yunnan	—	NC072346	NC072346
*Triplophysa orientalis*	—	—	NC030505	NC030505
*Triplophysa rongduensis*	Beipanjiang River, Guizhou	T22	PP204903	PP204903
*Triplophysa rosa*	Wujiang River, Chongqing	SWU 10100503	NC019587	NC019587
*Triplophysa sellaefer*	Juma River, Hebei	IHB 2015103	KY851112	KY851112
*Triplophysa stenura*	Yarlung Zangbo River, Tibet	—	NC032692	NC032692
*Triplophysa stoliczkai*	—	—	NC017890	NC017890
*Triplophysa weiheensis*	Weihe River, Gansu	—	NC086762	NC086762
*Triplophysa wudangensis*	Wujiang River, Guizhou	T26	PP204906	PP204906
*Triplophysa zhengfengens*	Beipanjiang River, Guizhou	GZNU 20180419002	NC063617	NC063617

## Results

### Taxonomic account

#### Claea tongziensis sp. nov.

Taxon classificationAnimaliaCypriniformesNemacheilidae

AE5881F7-E321-5055-8C2A-F9325C8514FA

https://zoobank.org/6A0AFB2F-AEE0-4393-8DEC-30C0A0E901DB

##### Type material.

**Figure 2.**
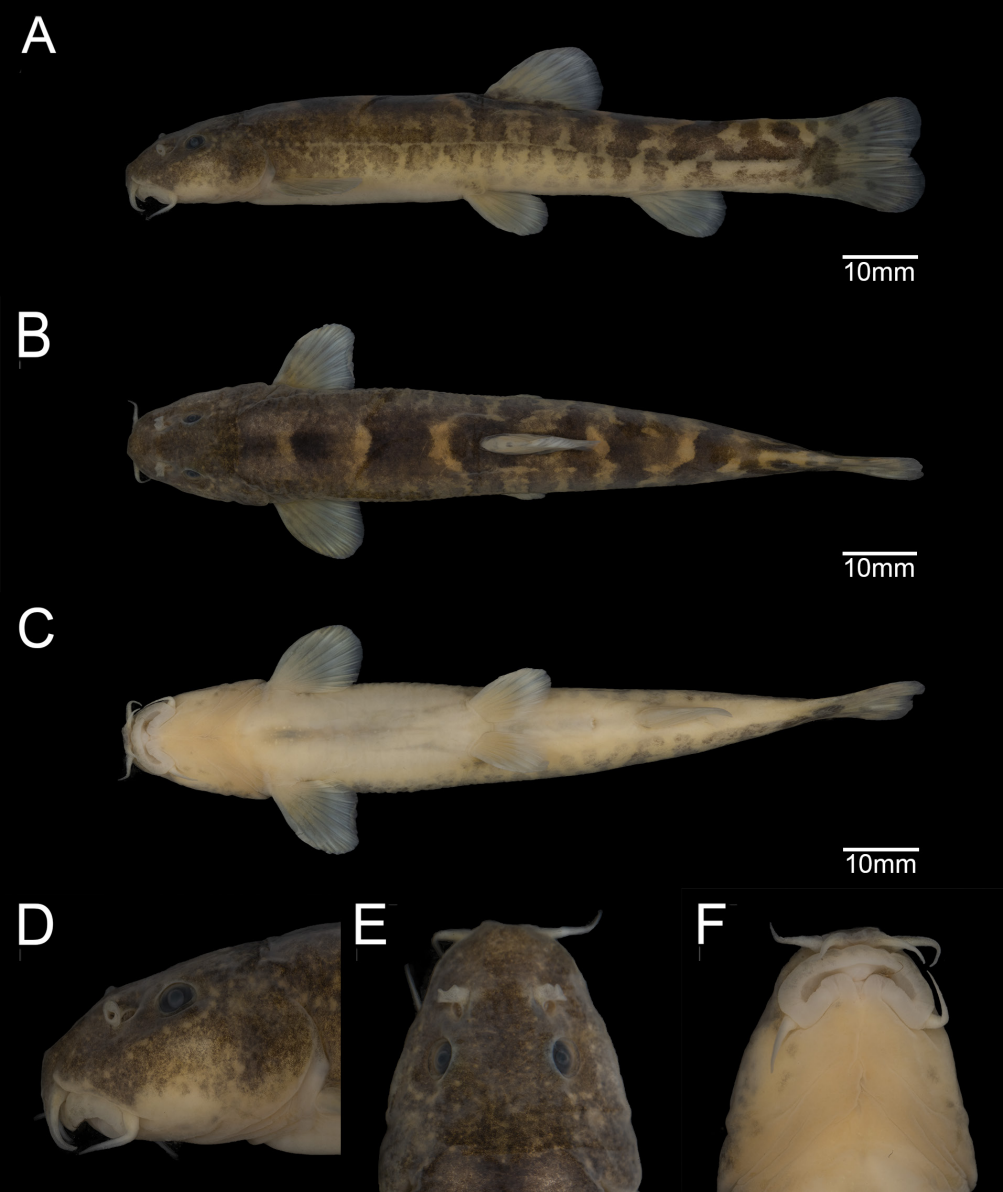
Morphological characteristics of holotype IHB 202405240001 of *Claea tongziensis* sp. nov. in 10% formalin preservative. **A**. Lateral view; **B**. Dorsal view; **C**. Ventral view; **D**. Lateral view of head; **E**. Dorsal view of head; **F**. Ventral view of head.

***Holotype***. IHB 202405240001 (Fig. 2), 87.3 mm SL; the headwaters of the Tongzi River in the Chishui River drainage, at Shiban Village, Mazong Miao Ethnic Township, Tongzi County, Guizhou Province, China (28.2235°N, 106.9331°E; 1055 m elevation; Fig. 1); collected by Fei Liu and Hai-Min Lv on 24 May 2024.

***Paratypes***. IHB 202405240002–12, 11 specimens, 54.4–72.8 mm SL; all other information same as holotype. IHB 202311020001–12, 12 specimens, 49.8–76.8 mm SL; collected on 2 November 2023, other information same as holotype.

##### Diagnosis.

The major diagnostic characters between *Claea tongziensis* sp. nov. and five known *Claea* species are provided in Table 2. *Claea tongziensis* sp. nov. can be distinguished from its congeneric species by the following combination of characters: (1) seven branched dorsal-fin rays (vs eight in *C. dafangensis*, *C. minibarba*, and *C. wulongensis*, and eight to nine in *C. scet*); (2) three unbranched anal-fin rays (vs two in *C. dabryi* and *C. minibarba*, and one in *C. wulongensis*). *Claea tongziensis* sp. nov. is further distinguished from *C. dabryi* by having shorter outer rostral barbels (18.9–29.3% HL vs 32.0–38.1% HL). *Claea tongziensis* sp. nov. is further distinguished from *C. scet* by having more vertebrae (4+40–42 vs 4+38), a prominent processus dentiformis (vs reduced), a more anteriorly situated pelvic fin (prepelvic length 46.9–51.7% SL vs 51.7–56.0% SL), a longer distance between pelvic-fin origin and anal-fin origin (23.8–28.6% SL vs 20.4–23.1% SL), a longer distance between posterior margin of anus and anal-fin origin (4.7–7.0% SL vs 2.5–3.5% SL), a pair of eyes of normal size (diameter 13.5–18.5% HL vs 4.0–5.9% HL). *Claea tongziensis* sp. nov. is further distinguished from *C. wulongensis* by having more vertebrae (4+40–42 vs 4+38–39), a narrower interorbital distance (22.2–29.6% HL vs 38.5–43.1% HL).

**Table 2.** Major diagnostic characters between *Claea tongziensis* sp. nov. and 5 known species of *Claea*.

**Diagnostic characters**	***Claea tongziensis* sp. nov. (*n* = 24)**	***Claea dabryi* (*n* = 11)**	***Claea dafangensis* (*n* = 14)**	***Claea minibarba* (*n* = 7)**	***Claea scet* (*n* = 7)**	***Claea wulongensis* (*n* = 9)**
Processus dentiformis	Prominent	Prominent	Prominent	Prominent	Reduced	Prominent
Dorsal-fin rays	iii, 7	iii, 7–9	iii, 8	iii, 8	iii, 8–9	iii, 8
Anal-fin rays	iii, 5–6	ii, 5–6	iii, 5	ii, 5–6	iii, 5–6	i, 5
Pectoral-fin rays	i, 7–9	i, 8–10	i, 8–9	i, 8–10	i, 10	i, 8–9
Pelvic-fin rays	i, 5–7	i, 6–7	i, 6	i, 5–6	i, 5–6	i, 5–7
Caudal-fin rays	2+15–17	2+14–16	16	2+15–16	2+16	2+16
Vertebrae	4+40–42	4+38–42	—	4+41–43	4+38	4+38–39
Prepelvic length (% SL)	46.9–51.7	46.1–47.6	40.3–49.8	43.6–48.9	51.7–56.0	48.3–50.9
Pelvic–anal distance (% SL)	23.8–28.6	24.4–26.8	22.4–32.8	22.5–26.9	20.4–23.1	21.6–26.9
Anus–anal distance (% SL)	4.7–7.0	4.9–6.7	—	4.2–7.8	2.5–3.5	4.0–7.2
Eye diameter (% HL)	13.5–18.5	13.5–17.2	10.9–21.0	15.3–19.0	4.0–5.9	11.1–19.1
Interorbital width (% HL)	22.2–29.6	23.4–26.4	23.6–39.0	27.5–31.6	25.7–31.9	38.5–43.1
Outer rostral-barbel length (% HL)	18.9–29.3	32.0–38.1	16.2–51.1	24.3–30.6	23.3–29.8	21.4–41.5

##### Description.

Morphometric data for 24 type specimens of *C. tongziensis* sp. nov. are presented in Table 3. Body elongated, anteriorly subcylindrical and posteriorly laterally compressed. Dorsal profile nearly straight on head, slightly convex along body anterior to dorsal-fin origin, and slightly concave posterior to dorsal fin. Dorsal profile deepest anterior to dorsal-fin origin, body depth 12.1–17.3% SL. Ventral profile almost flat from snout tip to anal-fin origin, and slightly concave posterior to anal-fin origin. Head slightly depressed, head width greater than depth (head width/head depth = 1.2–1.4). Snout moderately blunt; snout length almost always shorter than postorbital head length. Anterior and posterior nostrils proximally positioned; anterior nostril located in a nasal flap, short tubular, tip not extended into a barbel. Eyes well developed and dorsolaterally situated on head, diameter 13.5–18.5% HL. Mouth inferior and arched. Lips thick, smooth or slightly furrowed; lower lip with a distinct V-shaped median incision. Upper jaw with a median prominent processus dentiformis; lower jaw scoop-shaped. Three pairs of barbels present; inner rostral barbels not extending to mouth corner, length 15.7–23.5% HL; outer rostral barbels not extending to anterior margin of the eye, length 18.9–29.3% HL; maxillary barbels not extending to posterior margin of the eye, length 19.2–29.8% HL.

**Table 3.** Morphometric measurements for *Claea tongziensis* sp. nov.

**Characters**	**Holotype**	**Holotype + paratypes (*n =* 24)**		
		**Range**	**Mean**	**SD**
Standard length (mm)	87.3	49.8–87.3	61.4	8.8
Head length (mm)	19.3	11.6–19.3	13.6	1.8
In percentage of SL (%)				
Head length	22.1	20.2–23.8	22.2	0.9
Body depth	15.9	12.1–17.3	14.9	1.6
Body width	15.8	11.7–17.0	14.1	1.8
Predorsal length	52.3	51.4–57.2	54.1	1.4
Postdorsal length	37.6	35.8–41.7	38.5	1.4
Prepectoral length	20.8	19.9–24.2	22.2	1.0
Prepelvic length	48.1	46.9–51.7	48.7	1.1
Preanal length	72.3	70.6–77.1	73.8	1.4
Preanus length	66.5	65.0–70.8	67.9	1.3
Dorsal-fin length	17.6	15.4–20.4	18.2	1.3
Dorsal-fin base length	11.4	10.7–12.5	11.5	0.5
Pectoral-fin length	15.6	14.5–20.0	17.4	1.3
Pelvic-fin length	12.9	12.0–15.8	14.5	1.0
Anal-fin length	14.6	13.1–17.0	15.4	1.1
Anal-fin base length	7.6	7.5–9.3	8.4	0.5
Caudal-fin length	16.3	16.3–23.8	19.9	1.7
Caudal peduncle length (CPL)	19.0	16.4–20.2	18.1	1.0
Caudal peduncle depth (CPD)	10.4	8.2–11.5	9.7	1.1
Pectoral–pelvic distance	29.3	25.8–29.6	27.7	1.1
Pelvic–anal distance	24.7	23.8–28.6	26.2	1.0
Anus–anal distance	5.8	4.7–7.0	5.7	0.6
In percentage of HL (%)				
Head depth	54.3	45.6–58.0	51.4	3.1
Head width	73.7	58.0–74.1	66.3	4.4
Snout length	48.8	38.5–48.8	42.1	2.3
Mouth width	26.4	18.5–26.4	22.6	2.2
Eye diameter	13.5	13.5–18.5	16.1	1.2
Postorbital head length	41.4	41.4–48.3	45.2	1.8
Interorbital width	27.2	22.2–29.6	25.2	2.1
Internostril width	23.5	19.1–23.8	21.7	1.4
Inner rostral-barbel length	21.9	15.7–23.5	19.6	2.1
Outer rostral-barbel length	27.1	18.9–29.3	25.3	2.2
Maxillary-barbel length	28.2	19.2–29.8	25.2	2.6
CPD/CPL (%)	54.7	43.6–66.4	53.8	7.5

Dorsal-fin rays iii, 7; anal-fin rays iii, 5–6; pectoral-fin rays i, 7–9; pelvic-fin rays i, 5–7; 15–17 branched caudal-fin rays. Distal margin of dorsal fin truncate or slightly rounded, dorsal-fin origin posterior to pelvic-fin origin, predorsal length 51.4–57.2% SL. Pectoral fin rounded and moderately developed, with its tip extending past the midpoint between pectoral-fin origin and pelvic-fin origin but not reaching to pelvic-fin origin. Pectoral-pelvic distance slightly greater than pelvic-anal distance, tip of pelvic fin not reaching to anus. Anus closer to anal-fin origin than pelvic fin origin. Distal margin of anal fin truncate or convex, tip of anal fin not reaching to caudal-fin base. Posterior margin of caudal fin deeply concave, upper lobe equal to lower lobe in length.

**Figure 3.**
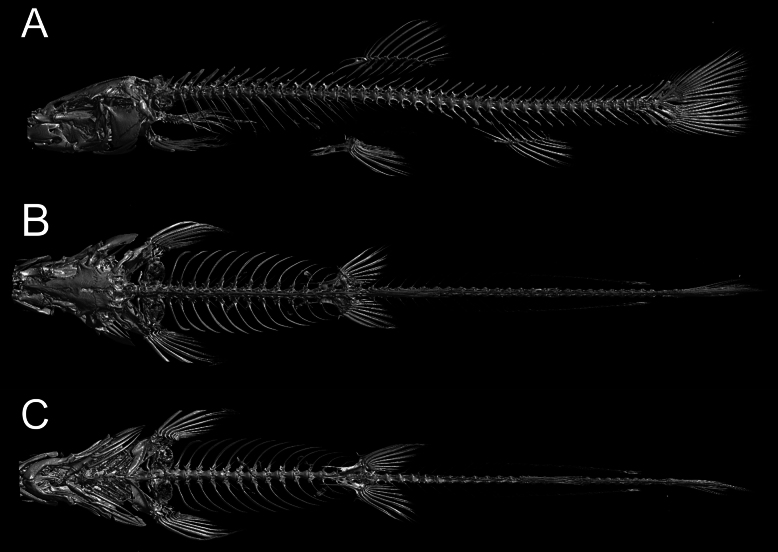
Micro-CT graph of the skeleton of holotype IHB 202405240001 of *Claea tongziensis* sp. nov. **A**. Lateral view; **B**. Dorsal view; **C**. Ventral view.

Body entirely smooth and scaleless. Cephalic lateral line system developed. Lateral line complete and almost straight. Intestine originating from posterior U-shaped stomach, extending straight to the anus without bends. Anterior chamber of swim bladder divided into two lateral chambers, connected by a short tube and enclosed in a bony capsule; posterior chamber completely degenerated (Fig. 3). Vertebrae 4+40–42 (four specimens).

##### Colouration.

**Figure 4.**
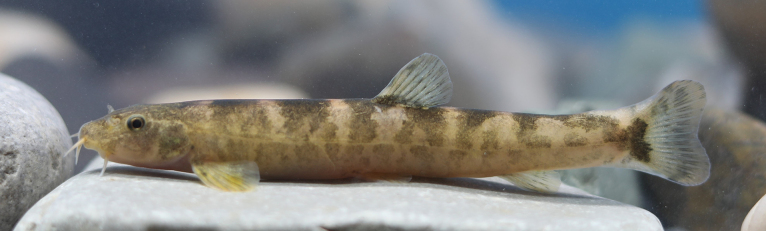
Living specimen of *Claea tongziensis* sp. nov.

In live specimens (Fig. 4), body yellowish dorsally and laterally, and slightly lighter ventrally. Irregular, dark-brown patches on flank, six to eight distinct, dark-brown, transverse saddles across dorsum. All fins yellowish white; one or two dark bars across dorsal fin and caudal fin; other fins covered with dark spots. Dark broad blotches on caudal-fin base. In 10% formalin-preserved specimens (Fig. 2), the patches on the body darkened.

##### Sexual dimorphism.

Sexual dimorphism was absent among the examined type specimens of *Claea tongziensis* sp. nov.

##### Distribution and habitat.

**Figure 5.**
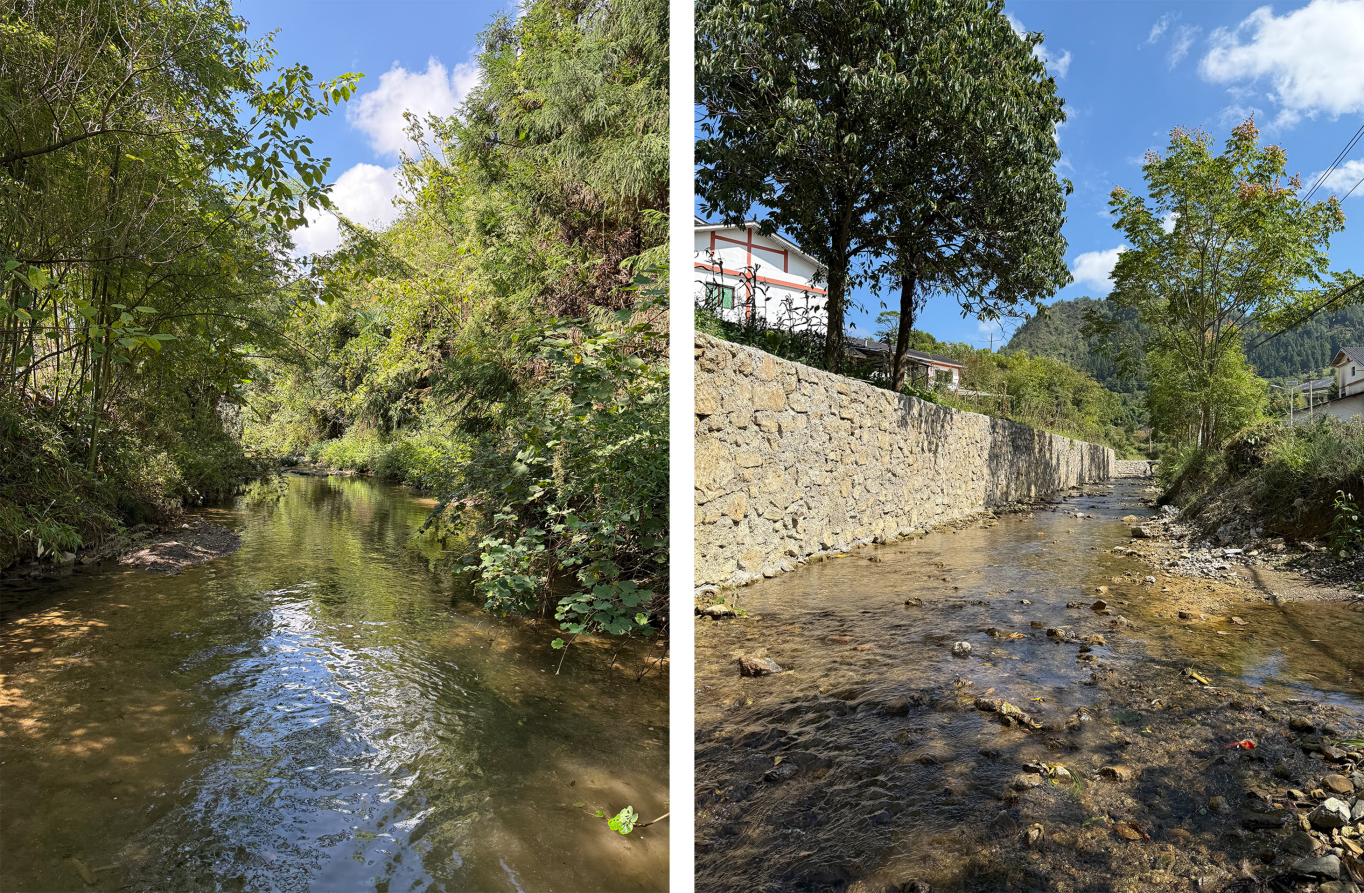
Habitat of the type locality of *Claea tongziensis* sp. nov.

This new species *Claea tongziensis* sp. nov. is presently only known from the headwaters of the Tongzi River, a tributary of the Chishui River in the upper Yangtze River drainage, at Shiban Village, Mazong Miao Ethnic Township, Tongzi County, Guizhou Province, China (Fig. 1). *Claea tongziensis* sp. nov. inhabits in flowing, clear mountain streams with gravel and cobble substrates (Fig. 5). Co-existing fish species are *Opsariichthys acutipinnis macrolepis* (Yang & Huang, 1964) and *Acrossocheilus yunnanensis* (Regan, 1904).

##### Etymology.

The specific epithet tongziensis is formed by combining “Tongzi”, the name of the county where type specimens were collected, with Latin adjectival suffix -*ensis*, denoting origin or locality. The scientific name directly refers to the type locality of the new species. The Chinese common name 桐梓山鳅 is proposed for the new species.

### Molecular analyses

**Figure 6.**
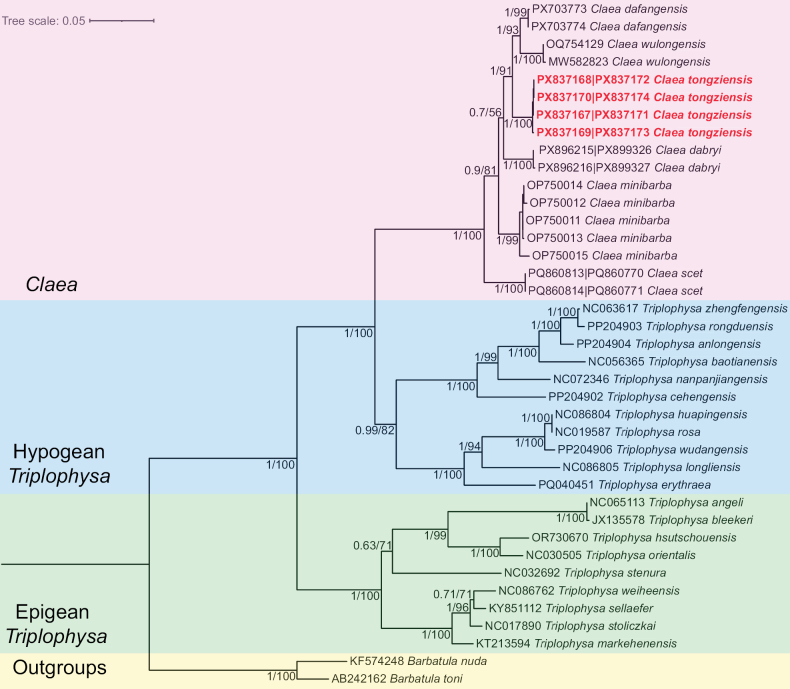
Phylogenetic tree based on *cytb* and *cox1* gene sequences. Bayesian posterior probabilities (BBP) from BI analysis and ultrafast bootstrap values (UBP) from ML analysis are shown beside nodes.

The phylogenetic trees of ML tree and BI tree based on *cytb* and *cox1* sequences displayed identical topologies. The ML tree is shown in Fig. 6. Four type specimens of the new species *Claea tongziensis* sp. nov. formed a strongly supported monophyletic group (BPP/UBP = 1/100) and a sister group with the lineage comprising *C. dafangensis* and *C. wulongensis*. Moreover, all species of *Claea* collectively formed a monophyletic group (BPP/UBP = 1/100) and were embedded within the genus *Triplophysa* as the sister group to hypogean *Triplophysa*. The mean K2P genetic distances between *C. tongziensis* sp. nov. and other species of the genus *Claea* ranged from 2.82% (with *C. dafangensis*) to 6.41% (with *C. scet*) based on *cytb* gene (Table 4).

**Table 4.** Mean K2P genetic distances among different species of the genus *Claea* based on *cytb* gene sequences.

**ID**	**Species**	**1**	**2**	**3**	**4**	**5**	**6**
1	*Claea tongziensis* sp. nov.	—					
2	*Claea dabryi*	0.0441	—				
3	*Claea dafangensis*	0.0282	0.0400	—			
4	*Claea minibarba*	0.0479	0.0466	0.0392	—		
5	*Claea scet*	0.0641	0.0648	0.0618	0.0630	—	
6	*Claea wulongensis*	0.0395	0.0475	0.0266	0.0459	0.0647	—

## Discussion

In conclusion, the validity of the new species is strongly supported by both morphological and molecular evidence. The description of the new species increases the number of species of *Claea* to six, among which three are epigean: *C. dabryi*, *C. minibarba*, and *C. tongziensis* sp. nov. *Claea tongziensis* sp. nov. is the first species of its genus found in the Chishui River drainage. It remains unclear whether *C. tongziensis* sp. nov. is distributed in the mainstream and other tributaries of the Chishui River and whether there are other species of the genus *Claea* in the Chishui River drainage. Therefore, a systematic ichthyological survey of the Chishui River is essential to study the diversity and evolutionary history of the genus *Claea* in the upper Yangtze River.

In our phylogenetic analysis, *Claea tongziensis* sp. nov. forms a sister group with the lineage comprising hypogean *C. dafangensis* and hypogean *C. wulongensis*, although the latter two lack typical cave adaptation characters. This relationship suggests that hypogean *Claea* may be a branch of epigean *Claea* formed after recent migration into the underground environment, with a short divergence time. This hypothesis is supported by the geological context: both the Chishui River drainage (occupied by *C. tongziensis* sp. nov.) and the Wujiang River drainage (inhabited by *C. dafangensis* and *C. wulongensis*) are characterized by well-developed karst landscapes. Thus, these two adjacent rivers may be connected by underground rivers, providing opportunities for gene flow among *Claea* populations from different river systems. Previous studies about *Sinocyclocheilus* Fang, 1936 have demonstrated that the origin of cave fishes may be attributed to colonization events from surface to subterranean habitats, and that underground river systems play an important role for fish dispersal and gene flow in karst regions (Zhao and Zhang 2009; Li et al. 2021). Furthermore, it is worth mentioning that the type localities of the three species, *C. tongziensis* sp. nov. versus *C. dafangensis* and *C. wulongensis* are separated by the Dalou Mountains, the watershed of the Chishui River and the Wujiang River. Although geological evidence indicates that the main body of the Dalou Mountains was formed in the late Cretaceous and experienced rapid uplift after the Miocene due to compression of the Qinghai-Tibet Plateau, it did not become the watershed between the Chishui River and the Wujiang River until the Pliocene–Pleistocene (5–1 Ma) (Zhou et al. 2016; Guo et al. 2021; Zhang et al. 2025). Therefore, the relatively close genetic relationship between *C. tongziensis* sp. nov. and the species pair formed by *C. dafangensis* and *C. wulongensis* may be related to the possible water connectivity between the two rivers prior to the formation of the watershed. In our study, epigean *C. dabryi* and hypogean *C. scet* exhibit a relatively distant genetic affinity, which is inconsistent with the finding reported by Lei et al. (2025). This discrepancy stems from the fact that the *C. dabryi* specimens used for molecular analysis in our study were collected from its type locality (Dong River, Baoxing County, Sichuan Province), whereas the *C. dabryi* specimens analysed by Lei et al. (2025) were only broadly reported to be from the Jinsha River. Although *C. dabryi* was reported widely distributed in the upper Yangtze River (Zhu 1989), we suppose that Jinsha River population may not be true *C. dabryi* but may represent a distinct, undescribed species or a surface ecotype closely allied to *C. scet*. Our finding reflects the importance of topotypic materials in species delimitation and reveals potential cryptic diversity within the genus *Claea*.

Our molecular phylogenetic analysis shows that *Claea* is nested with *Triplophysa*, clustering a subclade with hypogean *Triplophysa* as a sister group and occupying a basal position within this subclade. This is consistent with previous studies (Yan 2017; Zhang et al. 2024; Lei et al. 2025). In Yan’s (2017) study, *Triplophysa* diverged into the epigean *Triplophysa* taxa and taxa consisting of hypogean *Triplophysa* and *C. dabryi* in the early Miocene (approximately 17.92 Ma). We speculate that the hypogean *Triplophysa* and *Claea* may originate from the most recent common ancestor with cave adaptation potential. The *Claea* lineage dispersed to what is now the edge of the Yunnan-Guizhou Plateau, inhabiting surface streams and caves, and diverged first. In contrast, the hypogean *Triplophysa* lineage spread deeply into the interior of the Yunnan-Guizhou Plateau and differentiated rapidly driven by the development of karst landforms (Yan 2017; Zhang et al. 2024). Although Ren (2011) proposed that *Claea* might be placed within the genus *Triplophysa*, we argue that *Claea* should be recognized as an independent and valid genus. This argument is primarily grounded in two pieces of evidence: the morphological differences in processus dentiformis and sexual dimorphism between the two genera, and their sufficiently long divergence time. Future work requires prioritize confirming the taxonomic status of *Claea* through extensive sampling, comprehensive morphological comparisons, and integrative analysis of mitochondrial and nuclear gene markers.

During the fish survey of the Tongzi River in June 2025, we found that the river regulation works have caused drastic changes in the type locality of *Claea tongziensis* sp. nov. In addition, illegal fishing, sewage discharge, and alien invasion are also threatening *C. tongziensis* sp. nov. and other fishes. Therefore, ecological restoration and environmental conservation measures are urgently needed to protect these fishes and their habitats.

### Comparative materials

***Claea dabryi*** (*n* = 11): IHB uncatalogued, 51.8–73.2 mm SL; China: Sichuan Province: Baoxing County: Dong River (type ocality).

***Claea dafangensis*** (*n* = 14): Data from Wang et al. (2026); GZNU20190804001–6, GZNU20241114001–4, GZNU20241204001-4, 49.4–80.2 mm SL; China: Guizhou Province: Dafang County: Yuchong Town: Yuchong River (type locality).

***Claea minibarba*** (*n* = 7): IHB 202204288315–18, 47.7–63.1 mm SL; China: Hubei Province: Badong County: Yanduhe Town: Shennong-Xi (type locality). IHB 202111056757-59, 64.8–69.3 mm SL; China: Hubei Province: Jianshi County: Gaoping Town: Yesan-He.

***Claea scet*** (*n* = 7): IHB 202305300001-5, IHB 202305300008-9, 49.4–56.4 mm SL; China: Sichuan Province: Leshan City: Hulu Town: Taojin Cave (type locality).

***Claea wulongensis*** (*n* = 9): SWU 2019051301-9, 49.0–67.2 mm SL; China: Chongqing City: Wulong County: Furong Cave (type locality).

## Supplementary Material

XML Treatment for Claea tongziensis

## Additional information

### Artificial Intelligence (AI) use

The authors accept full responsibility for the content of the manuscript, including the disclosure of any use of AI.

No AI tools were used in the preparation of this manuscript.

### Author ORCIDs

Meng-Fei Zhao https://orcid.org/0009-0000-9271-1464

Hai-Min Lv https://orcid.org/0009-0009-7713-6022

Jun-Hao Huang https://orcid.org/0009-0004-0671-1003

Fei Liu https://orcid.org/0000-0003-4404-0971

### Data availability

All of the data that support the findings of this study are available in the main text.

## References

[B1] Bănărescu P, Nalbant TT (1976) The genus *Oreias* Sauvage, 1874 (Pisces, Cobitidae). Nymphaea 4: 185–193.

[B2] Cao WX, Wu XW (1962) An investigation of the fish biology and fishery problems in Ganze-Apa region of western Szechwan Province. Acta Hydrobiologica Sinica 3(2): 79–112. 10.3724/issn1000-3207-1962-2-79-4

[B3] Chen H, Zhang H, Chen YX, Freyhof J (2019) A review of the *Barbatula* loaches (Teleostei: Nemacheilidae) from north-eastern China, with the description of four new species. Zootaxa 4565(1): 1–36. 10.11646/zootaxa.4565.1.1 31716487

[B4] Chen HL, Li R, Zhang YS, Wu QL, Yuan J, Gao JY (2023) Comparison of ecosystem health in different geomorphic regions of Chishui River basin, southwest China. Chinese Journal of Applied Ecology 34(7): 1912–1922. [In Chinese] 10.13287/j.1001-9332.202307.024 37694475

[B5] Chen L, Lu ZM, Mao WN (2006) A new species of *Schistura* discovered in Yunnan, China. Guizhou Agricultural Sciences 34(5): 54–55. [In Chinese]

[B6] Chen SJ, Sheraliev B, Shu L, Peng ZG (2021) *Triplophysa wulongensis*, a new species of cave-dwelling loach (Teleostei, Nemacheilidae) from Chongqing, Southwest China. ZooKeys 1026: 179–192. 10.3897/zookeys.1026.61570 PMC8018939 33850421

[B7] Chu XL, Chen YR (1990) The Fishes of Yunnan, China. Part II. Science Press, Beijing, 313 pp. [In Chinese]

[B8] Du LN, Yang J, Min R, Chen XY, Yang JX (2021) A review of the Cypriniform tribe Yunnanilini Prokofiev, 2010 from China, with an emphasis on five genera based on morphologies and complete mitochondrial genomes of some species. Zoological Research 42(3): 310–334. 10.24272/j.issn.2095-8137.2020.229 PMC8175957 33929106

[B9] Guo WX, Tang JM, Ouyang JS, Wang T, Wang X, Wang Y (2021) Characteristics of structural deformation in the southern Sichuan Basin and its relationship with the storage condition of shale gas. Natural Gas Industry 41(5): 11–19. [In Chinese]

[B10] Kalyaanamoorthy S, Minh BQ, Wong TK, Von Haeseler A, Jermiin LS (2017) ModelFinder: fast model selection for accurate phylogenetic estimates. Nature Methods 14(6): 587–589. 10.1038/nmeth.4285 PMC5453245 28481363

[B11] Katoh K, Standley DM (2013) MAFFT multiple sequence alignment software version 7: improvements in performance and usability. Molecular Biology and Evolution 30(4): 772–780. 10.1093/molbev/mst010 PMC3603318 23329690

[B12] Kimura M (1980) A simple method for estimating evolutionary rates of base substitutions through comparative studies of nucleotide sequences. Journal of Molecular Evolution 16(2): 111–120. 10.1007/bf01731581 7463489

[B13] Kottelat M (1990) Indochinese nemacheilines: A revision of nemacheiline loaches (Pisces: Cypriniformes) of Thailand, Burma, Laos, Cambodia and southern Viet Nam. Dr Friedrich Pfeil, Munich, 262 pp.

[B14] Kottelat M (2011) *Claea*, a new replacement name for *Oreias* Sauvage, 1874 (Teleostei: Nemacheilidae). Ichthyological Exploration of Freshwaters 21(4): 384.

[B15] Kottelat M (2012) Conspectus cobitidum: an inventory of the loaches of the world (Teleostei: Cypriniformes: Cobitoidei). The Raffles Bulletin of Zoology 26: 1–199.

[B16] Kumar S, Stecher G, Tamura K (2016) MEGA7: Molecular Evolutionary Genetics Analysis version 7.0 for bigger datasets. Molecular Biology and Evolution 33(7): 1870–1874. 10.1093/molbev/msw054 PMC8210823 27004904

[B17] Lei HT, He L, Huang JH, Zhou JJ, He DK (2025) Description of a new cave-dwelling species of *Claea* (Teleostei, Cypriniformes, Nemacheilidae) from the Yangtze River basin in Sichuan, southern China. Zoosystematics and Evolution 101(2): 681–695. 10.3897/zse.101.146469

[B18] Letunic I, Bork P (2024) Interactive Tree of Life (iTOL) v6: recent updates to the phylogenetic tree display and annotation tool. Nucleic Acids Research 52(W1): W78–W82. 10.1093/nar/gkae268 PMC11223838 38613393

[B19] Li GW, Xiao NW, Li JS (2021) Analysis the trend of ecosystem quality based on ideal reference key parameters in the Chishui River Basin, China. Acta Ecologica Sincia 41(18): 7114–7124. 10.5846/stxb202103040590 [In Chinese]

[B20] Li RH, Wang XA, Bian C, Gao ZJ, Zhang YW, Jiang WS, Wang M, You XX, Cheng L, Pan XF, Yang JX, Shi Q (2021) Whole-genome sequencing of *Sinocyclocheilus maitianheensis* reveals phylogenetic evolution and immunological variances in various *Sinocyclocheilus* Fishes. Frontiers in Genetics 12: 736500. 10.3389/fgene.2021.736500 PMC8523889 34675964

[B21] Liao JW, Wang DZ, Luo ZF (1997) A new species and a new subspecies of *Schistura* from Guangxi and Guizhou, China (Cypriniformes: Cobitidae: Noemacheilinae). Acta Academiae Medicinae Zunyi 20(2–3): 4–7.

[B22] Liu F, Wang J, Zhang FB, Liu HZ, Wang JW (2020) Spatial organisation of fish assemblages in the Chishui River, the last free‐flowing tributary of the upper Yangtze River, China. Ecology of Freshwater Fish 30(1): 48–60. 10.1111/eff.12562

[B23] Liu SQ, Mayden RL, Zhang JB, Yu D, Tang QY, Deng X, Liu HZ (2012) Phylogenetic relationships of the Cobitoidea (Teleostei: Cypriniformes) inferred from mitochondrial and nuclear genes with analyses of gene evolution. Gene 508(1): 60–72. 10.1016/j.gene.2012.07.040 22868207

[B24] Minh BQ, Nguyen MA, Von HA (2013) Ultrafast approximation for phylogenetic bootstrap. Molecular Biology and Evolution 30(5): 1188–1195. 10.1093/molbev/mst024 PMC3670741 23418397

[B25] Nguyen LT, Schmidt HA, Von HA, Minh BQ (2015) IQ-TREE: a fast and effective stochastic algorithm for estimating maximum-likelihood phylogenies. Molecular Biology and Evolution 32(1): 268–274. 10.1093/molbev/msu300 PMC4271533 25371430

[B26] Nichols JT (1944) The fresh-water fishes of China. In: Tyler R (Ed.) Natural History of Central Asia, Volume IX. The American Museum of Natural History, New York, [xxxvi +] 322 pp. [10 pls]. 10.5962/bhl.title.12103

[B27] Peng ZG (2005) Phylogenetic relationships of Eurasian catfishes (Otocephala: Siluriformes) and divergence time estimates for major otocephalan clades. Doctoral thesis, Institute of Hydrobiology, Chinese Academy of Sciences, Wuhan. [In Chinese]

[B28] Ren Q (2011) Phylogeography and morphology of regional subgroups of *Triplophysa*. Master thesis, Kunming Institute of Zoology, Chinese Academy of Sciences, Kunming. [In Chinese]

[B29] Ronquist F, Teslenko M, Van DMP, Ayres DL, Darling A, Höhna S, Larget B, Liu L, Suchard MA, Huelsenbeck JP (2012) MrBayes 3.2: efficient Bayesian phylogenetic inference and model choice across a large model space. Systematic Biology 61(3): 539–542. 10.1093/sysbio/sys029 PMC3329765 22357727

[B30] Sauvage HE (1874) Notices ichthyologiques. II. Sur un cyprin de genre nouveau provenant de Chine. Revue et Magasin de Zoologie 2(3): 332–340.

[B31] Wang JJ, Luo T, Xiao MY, Xie X, Wang YL, Deng HQ, Xiao N, Zhou J (2026) *Claea dafangensis* (Cypriniformes, Nemacheilidae), a new cave-dwelling fish from the upper Wujiang River, Guizhou Province, China. Zoosystematics and Evolution 102(2): 519–531. 10.3897/zse.102.172325

[B32] Wu J, Wang J, He Y, Cao W (2011) Fish assemblage structure in the Chishui River, a protected tributary of the Yangtze River. Knowledge and Management of Aquatic Ecosystems 400: 11. 10.1051/kmae/2011023

[B33] Xiang CY, Gao F, Jakovlić I, Lei HP, Hu Y, Zhang H, Zou H, Wang GT, Zhang D (2023) Using PhyloSuite for molecular phylogeny and tree-based analyses. IMeta 2(1): e87. 10.1002/imt2.87 PMC10989932 38868339

[B34] Xiao WH, Zhang YP, Liu HZ (2001) Molecular systematics of *Xenocyprinae* (Teleostei: Cyprinidae): taxonomy, biogeography, and coevolution of a special group restricted in East Asia. Molecular Phylogenetics and Evolution 18(2): 163–173. 10.1006/mpev.2000.0879 11161753

[B35] Yan YL (2017) The origin and evolution of cave-dwelling group of *Triplophysa* fishes (Teleostei, Cypriniformes, Nemacheilidae). Master thesis, Southwest University, Chongqing. [In Chinese]

[B36] Zhang CY, Luo P, Huang F, Zhang E (2024) Revision of the loach genus *Claea* Kottelat, 2010 (Teleostei: Nemacheilidae) in China, with a description of a new species from the Chang-Jiang basin. Zootaxa 5543(3): 404–422. 10.11646/zootaxa.5543.3.6 39646098

[B37] Zhang D, Gao F, Jakovlić I, Zou H, Zhang J, Li WX, Wang GT (2020) PhyloSuite: an integrated and scalable desktop platform for streamlined molecular sequence data management and evolutionary phylogenetics studies. Molecular Ecology Resources 20(1): 348–355. 10.1111/1755-0998.13096 31599058

[B38] Zhang HS, Li ZW, Li JX, Tong K, Zheng ZY (2025) Meso‐Cenozoic Multiple Deformations of the Southern Margin of Sichuan Basin and Their Response to Tectonic Events in East Asia. Geological Journal 0: 1–19. 10.1002/gj.5221

[B39] Zhao YH, Zhang CG (2009) Endemic Fishes of *Sinocyclocheilus* (Cypriniformes: Cyprinidae) in China—Species Diversity, Cave Adaptation, Systematics and Zoogeography. Science Press, Beijing. [In Chinese]

[B40] Zhou LF, Chen KQ, Tang Y, Yang JG (2016) Tectonic deformation in Qijiang-Chishui area of south Sichuan since Late Yanshanian. Geological Science and Technology Information 35(4): 66–73. [In Chinese]

[B41] Zhu SQ (1989) The Loaches of the Subfamily Nemacheilinae in China (Cypriniformes: Cobitidae). Jiangsu Science and Technology Publishing House, Nanjing, 150 pp. [In Chinese]

[B42] Zhu SQ, Cao WX (1988) Descriptions of two new species and a new subspecies of Nemacheilinae from Yunnan Province (Cypriniformes: Cobitidae). Acta Zootaxonomica Sinica 13(1): 95–100. [In Chinese]

